# Individual Variations in Nucleus Accumbens Responses Associated with Major Depressive Disorder Symptoms

**DOI:** 10.1038/srep21227

**Published:** 2016-02-16

**Authors:** Masaya Misaki, Hideo Suzuki, Jonathan Savitz, Wayne C. Drevets, Jerzy Bodurka

**Affiliations:** 1 Laureate Institute for Brain Research, Tulsa, OK, United States; 2 Faculty of Community Medicine, University of Tulsa, Tulsa, OK, United States; 3 Janssen Pharmaceuticals, LCC, of Johnson & Johnson, Inc., Titusville, NJ, USA; 4 Center for Biomedical Engineering, University of Oklahoma, Norman, OK, United States; 5 College of Engineering, University of Oklahoma, Norman, OK, United States

## Abstract

Abnormal reward-related responses in the nucleus accumbens (NAcc) have been reported for major depressive disorder (MDD) patients. However, variability exists in the reported results, which could be due to heterogeneity in neuropathology of depression. To parse the heterogeneity of MDD we investigated variation of NAcc responses to gain and loss anticipations using fMRI. We found NAcc responses to monetary gain and loss were significantly variable across subjects in both MDD and healthy control (HC) groups. The variations were seen as a hyperactive response subtype that showed elevated activation to the anticipation of both gain and loss, an intermediate response with greater activation to gain than loss, and a suppressed-activity with reduced activation to both gain and loss compared to a non-monetary condition. While these response variability were seen in both MDD and HC subjects, specific symptoms were significantly associated with the right NAcc variation in MDD. Both the hyper- and suppressed-activity subtypes of MDD patients had severe suicidal ideation and anhedonia symptoms. The intermediate subjects had less severity in these symptoms. These results suggest that differing propensities in reward responsiveness in the NAcc may affect the development of specific symptoms in MDD.

Major depressive disorder (MDD) is a heterogeneous disease associated with multiple symptoms and showing large variability across patients. The Diagnostic and Statistical Manual of Mental Disorders (DSM) assigns the diagnosis of MDD to patients with heterogeneous clinical syndromes^1^. Multiple symptom factors including depressed mood, anhedonia, psychomotor symptoms, and somatic symptoms, are characteristic of MDD^2^, perhaps explaining why no single biomarker meets the clinically useful levels of specificity and sensitivity^3^. This heterogeneity needs to be accounted for in attempts to elucidate the neurobiological basis of MDD^4^,^5^.

Abnormalities in reward processing are considered a major component of MDD pathology^6^,^7^,^8^. Anhedonia is one of the two core symptoms, along with depressed mood, in MDD^9^. Abnormal reward-related behaviors in various probabilistic learning and decision-making tasks^10^,^11^,^12^,^13^ as well as fMRI-measured brain activation in reward-processing regions such as the nucleus accumbens (NAcc), the striatum, and the medial prefrontal cortex have been reported in MDD^14^,^15^,^16^,^17^,^18^,^19^,^20^,^21^,^22^,^23^,^24^. Although abnormalities of reward processing have consistently been reported for MDD, the degree of dysfunction varies across studies^8^,^25^,^26^. Several studies showed reduced neural activity during the anticipation of reward^16^,^18^, while others showed reduced activity during receipt of rewards^14^,^15^,^27^ or in both anticipation and receipt of reward^19^,^20^,^22^. There is also another report^21^ showed no difference at both anticipation and acceptance of reward within the NAcc. This variability could be due to heterogeneity of MDD subjects. Indeed recent work^28^ reports two types of MDD subjects with higher and lower activity after reward receipt compared to healthy control (HC) in the ventral tegmental area (VTA) and the ventral striatum.

The heterogeneity of MDD patients imposes limitations on the standard study approach, in which subjects are classified into case (MDD) and control groups based on their diagnoses and/or symptom measures and then a difference between the groups is examined. While some studies carefully selected the case subjects based on specific criteria (e.g. anhedonia), symptom-based selection does not necessarily ensure patient group homogeneity. As anhedonia is not a specific symptom in MDD, but can also be seen in schizophrenia, substance abuse disorders, Parkinson’s disease, and over-eating patients^9^, identical symptoms are likely associated with different forms of neuropathology. For instance, distinct brain abnormalities have been reported in depressed and schizophrenia patients with anhedonia^27^.

Moreover, the control group may also be heterogeneous. Individual variability in activation of the NAcc has been reported for healthy subjects^29^,^30^,^31^. Deficits in reward-related brain responses similar to those reported in MDD have also been found in remitted MDD subjects^32^,^33^ and psychiatrically-healthy subjects with a parental history of depression^34^,^35^,^36^. A twins study^37^ indicated that more than 46% of reward responsiveness could be explained by genetic factor, which suggest reward responsiveness may reflect a genetically-influenced trait that is distributed across the population.

Heterogeneity in both MDD and HC groups and possible variability of associated neuropathology suggest that diagnosis- or symptom-based classification and comparison of group averages can limit elucidation of the neurobiological basis of MDD. Studies focusing on group average differences could have missed important information residing in individual variability, which might be able to explain heterogeneous symptoms in MDD. In this study, we focused on individual variability across subjects within a healthy and depressed group and its association with depression symptoms. The importance of investigating individual variability within a diagnostic group has been raised in the Research Domain Criteria (RDoC) project of the National Institute of Mental Health (NIMH) Strategic Plan^5^. RDoC suggested a dimensional approach, which examines the full range of variation from normal to abnormal among the fundamental functional components^4^,^5^. In line with the RDoC dimensional approach, our study identified heterogeneity of reward-related brain activation independent of diagnosis and symptomatology.

Specifically, we extracted subtypes of fMRI-measured NAcc activations to reward and punishment using an unsupervised classification analysis for MDD and HC subjects together. The extracted subtypes were then used as a reference to extract symptom factors that correlated with the derived phenotypic subtypes. We coined this approach as “Unsupervised Brain Subtyping and Symptoms Association”. Importantly, this approach not only directly identifies neurobiological variability without reference to diagnosis and symptomatology, but also allows for a bottom-up approach to understanding neurobiology instead of a top-down symptom-based search for the neurobiological correlates of interest.

Here we focus on the NAcc activation during the anticipation of reward and punishment. The NAcc, comprising part of the ventral striatum, is a critical component of the dopaminergic reward evaluation circuit in the brain^26^. Although the blunted activation to reward has been reported in both anticipation and acceptance of rewards, behavioral experiment indicated that MDD with anhedonia showed reduced wanting (expecting) of reward but no difference in liking (acceptance) of reward compared to HC^17^. Animal models of depression also indicated that VTA-NAcc dopaminergic circuit represents prediction or anticipation of reward^25^,^38^,^39^. Taken together, we hypothesized that reward-processing abnormality in the NAcc for MDD is characterized by its activation in reward anticipation more than in reward acceptance^40^.

We first examined variation of NAcc activation to anticipating monetary gains and losses across MDD and HC subjects using principal component analysis (PCA). We then applied a clustering analysis to the NAcc responses to describe the variation of response patterns. The extracted clusters were associated with symptom scores using a linear discriminant analysis (LDA). LDA was used to extract symptom subspace that was correlated with the NAcc response variation.

## Results

### Demographic and symptom rating

Table 1 shows subjects’ demographics and symptom ratings. Gender composition and mean age did not differ significantly between groups. The mean scores on all four symptom rating scales (sum scores for each scales) were significantly higher for MDD than HC by linear mixed-effect model (LMM) analyses^41^. For the SHAPS score, one HC and two MDD subjects who had missing values in some items were excluded from the statistical analysis of the SHAPS in Table 1.

### Diagnostic group average of NAcc response

We employed a monetary incentive delay (MID) task^42^ to measure NAcc response to monetary gains and losses. Supplementary Fig. S1 shows procedures of the task. NAcc responses during the anticipation (delay period in Supplementary Fig. S1) and outcome (feedback period in Supplementary Fig. S1) were evaluated separately. Average responses in the left and right NAcc regions for each of four monetary conditions (−$1.0, −$0.25, +$0.25, +$1.0) contrasted to the non-monetary condition ($0) were calculated in each subject.

**Figure 1:**
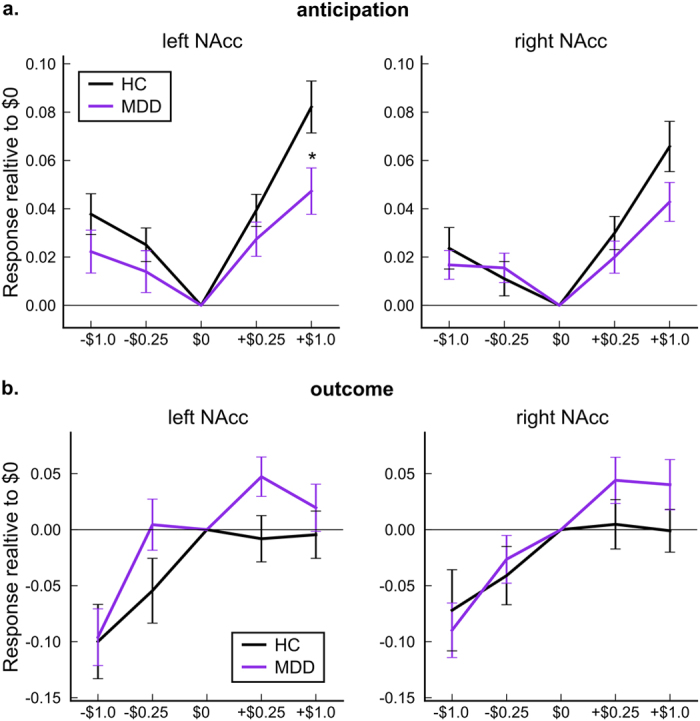
Group average of the left and right NAcc responses with standard errors of mean ((**a**) anticipation, (**b**) outcome periods), for HC and MDD subjects. A significant difference between groups was only seen at the + $1.0 condition in the left NAcc (^*^P < 0.05 by Tukey’s multiple comparison test).

Figure 1 shows group averages of NAcc responses for MDD and HC. LMM analysis with fixed effects of diagnosis, monetary condition, and their interaction along with age and gender was performed. Although the main effects of the monetary condition in both anticipation and outcome periods (*P* < 0.001 for both left and right NAcc) were significant, main effect of diagnosis was not significant (*P* = 0.325 and *P* = 0.405 for left and right NAcc, respectively. See Supplementary Table S1 for comprehensive statistics). Interaction between condition and diagnosis was significant for the left NAcc (*P* = 0.043) and post-hoc analysis (Tukey’s test) indicated that MDD had significantly lower activity at the + $1.0 condition in the left NAcc (*P* = 0.013 corrected).

### NAcc response variation

**Figure 2:**
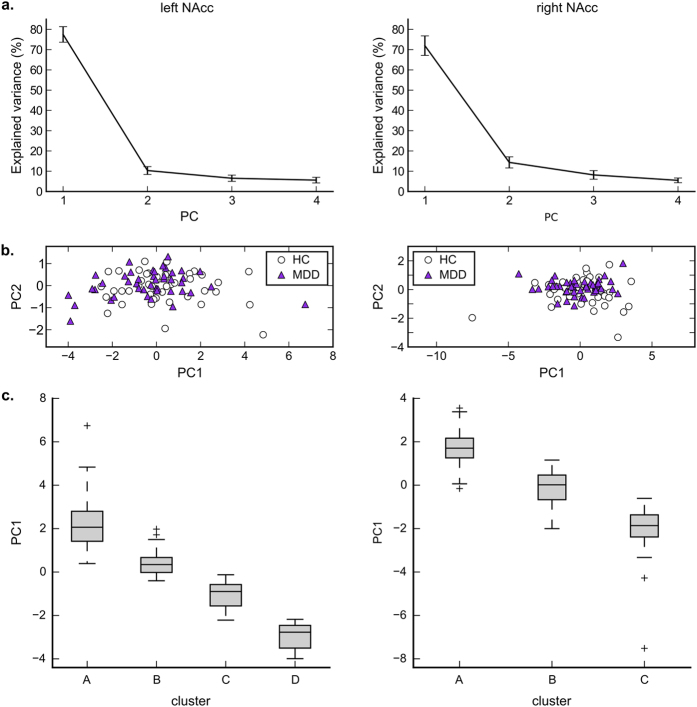
Percentage of explained variance in principal component analysis (**a**), distribution of principal scores in the first (PC1) and the second (PC2) principal components (**b**), and distribution of the first principal component score (PC1) across clusters.

Figure 2 shows results of PCA for the left and right NAcc response patterns in the anticipation period. Figure 2a shows percentage of explained variance with standard error of mean, and Fig. 2b shows distribution of principal scores in the first and the second principal components evaluated with leave-one-out cross-validation. More than 70% of variation was explained by the first principal component. The distribution of the first and the second principal scores for MDD and HC groups were highly overlapped.

To examine the response variation even further, we performed a clustering analysis on the NAcc activation patterns. Clustering was applied to four-dimensional vectors of response pattern (two loss and two gain conditions contrasted with the non-monetary condition). Similarity of NAcc activation between subjects was calculated by Euclidian distance between vectors of response pattern. We note that the responses to gain and loss were extracted as separate dimensions so that if there were unique difference either in gain or loss anticipation, which was suggested by a previous report for MDD^43^, the analysis could extract it.

Supplementary Fig. S2 shows a dendrogram of the clustering result for the NAcc response patterns and the process of cluster extraction. Four clusters for the left and three clusters for the right NAcc were extracted using the Bayesian information criterion (see Supplementary Fig. S2 for details). Figure 2c shows distribution of the first principal scores for each cluster. This indicated that cluster divisions were aligned to the first principal axis, which is not surprising considering majority of variance across subjects was explained by the first principal axis.

Left and right subtypes were highly overlapped (Supplementary Table S2) and association between the left and right NAcc subtypes were significant by a chi-square test (χ^2^(6) = 56.149, *P* < 0.001). Although the left and right subtypes were overlapped, there were still dissociations between them and hemispheric asymmetry of brain pathology in MDD has often been reported^44^, so we reported the results of left and right subtypes separately.

Table 2 shows the results of LMM analysis for subtype difference in the NAcc responses. Main effects of subtype and monetary condition and their interaction were significant (*P* < 0.001). Diagnosis effect was not significant except the three-way interaction of subtype × condition × diagnosis in the right NAcc (*P* = 0.002). Post-hoc analysis revealed that the difference between HC and MDD was significant only for the −$0.25 condition in subtype A of the right NAcc (MDD > HC, *P* = 0.009). Comprehensive post-hoc test results are shown in Supplementary Tables S3, S4, S5, and S6 for the anticipation and outcome periods of the left and right NAcc, respectively.

**Figure 3:**
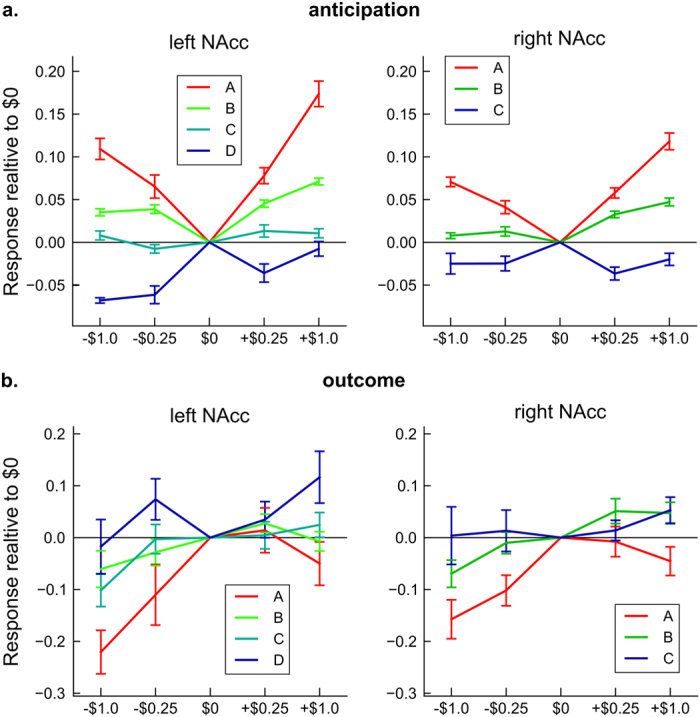
Average NAcc responses for each subtype in anticipation period (**a**) and outcome period (**b**) with standard errors of mean.

Figure 3 shows average response patterns for each subtype. In the left NAcc, subtype A showed significantly higher increases in the hemodynamic response during the anticipatory period to both gain and loss trials relative to the $0 (*P* < 0.001, Supplementary Table S3a). This subtype showed reversed response for the loss condition in the outcome period. Response to the −$1.0 outcome was significantly reduced compared to the $0 (*P* < 0.001, Supplementary Table S4a). Subtype B also showed significant increase of hemodynamic response to anticipated gains and losses (*P* < 0.001, Supplementary Table S3b) being more active to anticipated gains than losses (*P* < 0.001, Supplementary Table S3b). This subtype showed insensitivity to the differing outcome levels to gain and loss (Supplementary Table S4b). Subtype C showed insensitivity to the differing incentive levels of both the gain and loss trials in the anticipation periods (Supplementary Table S3c). This subtype showed significantly reduced response to the −$1.0 outcome relative to the $0 (*P* = 0.002, Supplementary Table S4c). Subtype D manifested significantly lower responses during the anticipation of both gains and losses relative to the $0 (*P* < 0.05, Supplementary Table S3d) except for the + $1.0 condition (*P* = 0.952, Supplementary Table S3d). This subtype showed insensitivity to the differing outcome levels (Supplementary Table S4d).

In the right NAcc, subtype A showed significantly higher response in anticipation of both gains and losses relative to the $0 condition (*P* < 0.001, Supplementary Table S5a). This subtype showed significantly reduced response to the loss outcomes relative to the $0 (*P* < 0.005, Supplementary Table S6a). Subtype B showed moderate activity to gains (*P* < 0.001, Supplementary Table S5b) but less response to losses. They showed significantly reduced response to the loss outcome (−$1.0) relative to the $0 condition (*P* = 0.014, Supplementary Table S6b). Subtype C showed significantly reduced response to both anticipated gains and losses compared to the $0 condition (Supplementary Table S5c). They showed insensitivity to the differing outcome levels to gain and loss (Supplementary Table S6c).

**Figure 4:**
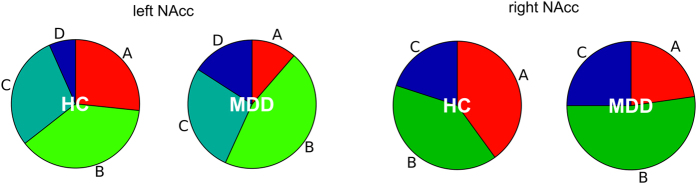
Proportions of NAcc subtypes in HC and MDD.

Figure 4 shows the proportions of the HC and MDD groups composing each subtype. The relative proportions of each subtype did not differ significantly between MDD and HC (χ^2^(3) = 4.755, *P* = 0.191 and χ^2^(2) = 3.085, *P* = 0.214, for the left and right NAcc, respectively by chi-square test). Gender composition, socioeconomic status (Hollingshead Four Factor Index of Socioeconomic Status), and behavioral responses in the MID task (reaction time, hit rate, and total earned money) were not significantly different between subtypes (see supplementary Table S7 for comprehensive statistical test results). Significant difference between subtypes was found in mean age (supplementary Table S7b). Post-hoc test showed that in the left NAcc subtype C was older than subtype B (*P* = 0.018) and in the right NAcc subtype C was older than subtype A (*P* = 0.015). For the MDD subjects, difference in total scores of each symptom scale, number of depressed episodes, and years since the first episode were not significantly different between subtypes (Supplementary Table S7g-l).

### Symptoms associated with the NAcc subtypes

Although the variability in the NAcc response patterns are not distinctive between MDD and HC, it might nevertheless be depression-related since previous studies have demonstrated that on average NAcc activation differs between healthy and MDD groups in response to rewarding stimuli^14^,^16^,^19^,^27^. Here we investigated symptom factors that could be related to NAcc response subtypes.

Symptoms associated with the NAcc response subtypes were extracted using linear discriminant analysis (LDA). Our purpose of using LDA is not classification but to extract symptom items that characterize the difference between NAcc subtypes. Fifty-nine symptom variables from the Hamilton rating scale for depression (HAM-D)^45^, the Hamilton anxiety rating scale (HAM-A)^46^, the Montgomery-Asberg Depression Rating Scale (MADRS)^47^, and the Snaith-Hamilton Pleasure Scale (SHAPS)^48^ were used as independent variables and the subtypes of NAcc response was used as dependent variable in the LDA. To extract critical symptom items that characterize the NAcc subtypes and to compensate for over-fitting problem in LDA, we used shrinkage discriminant analysis (SDA)^49^ combined with recursive feature elimination (RFE)^50^. This analysis was performed only for the MDD subjects since there were no or very small variances in these symptom scores for the HC group.

Supplementary Fig. S3 shows the history of recursive feature elimination and the distribution of the discriminant scores in the extraction of symptom items associated with the NAcc subtypes (See Methods for detailed procedure). The best classification score with a leave-one-out cross-validation was achieved with a set of 25 variables for the left and with nine variables for the right NAcc subtypes. Table 3 lists the selected symptom items and correlations between each score and the discriminant function output. We performed LMM analyses on the symptom items that significantly correlated with the subtype discriminant functions after Bonferroni correction.

**Figure 5:**
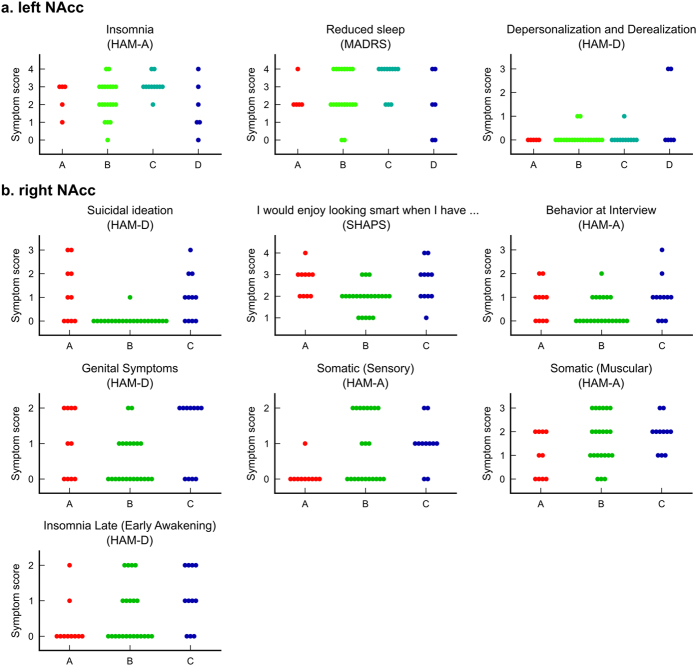
Distributions of symptom scores for each subtype in the left (**a**) and right (**b**) NAcc. Each point indicates a subject.

Figure 5 shows the distribution of symptom scores extracted for the analysis. For the left NAcc subtypes, LMM analyses with subtype, age, and gender as fixed effects showed significant main effect of subtype on ‘Depersonalization and Derealization (HAM-D)’ (*F*(3,38) = 3.394, *P* = 0.028) and ‘Insomnia (HAM-A)’ (*F*(3,38) = 3.086, *P* = 0.039). However, neither relationship remained significant after correcting for the number of LMMs performed in this step.

For the right NAcc subtypes, significant main effect of the subtype were seen in ‘Suicidal Ideation (HAM-D)’ (*F*(2,39) = 5.887, *P* = 0.005), one anhedonia item, ‘I would enjoy looking smart (SHAPS)’ (*F*(2,39) = 5.855, *P* = 0.006), and ‘Somatic (Sensory) (HAM-A)’ (*F*(2,39) = 4.348, *P* = 0.020). Only the relationships between suicidal ideation and the anhedonia item remained significant after correcting for the number of LMMs performed.

## Discussion

We investigated variability of NAcc responses to the anticipation of monetary gains and losses for MDD and HC subjects. Variations with either exaggerated or attenuated hemodynamic responses were observed among both healthy and depressed subjects without significant differences in the proportions of the subtypes between groups. The MDD and HC groups did, however, differ in their response to anticipating the largest potential monetary gain condition in the left NAcc on group average. This result is consistent with previous reports showing blunted ventral striatal activity during anticipation of monetary gains in MDD relative to HCs^16^, though there is considerable variability across studies. This variability may reflect biological heterogeneity within the population meeting DSM criteria for MDD and is consistent with our findings.

Importantly, while we observed the blunted NAcc response to gain anticipation for the average of MDD compared to the average of HC, we also found that the distribution of the NAcc response patterns to gain and loss anticipations were highly overlapped (Fig. 2b) and the response type variability was not significantly different between MDD and HC groups (Fig. 4). This indicated that population inference, such as a statistically significant difference between group averages does not necessarily suggest that NAcc activity to gain and loss in the context of the MID task is a sensitive marker for distinguishing individuals of MDD subjects from HC subjects.

Variability in the responsiveness to reward and loss also has been reported in healthy subjects^29^,^30^,^31^ and the results from several studies suggest that brain responsiveness to rewarding stimuli is a heritable factor^34^,^35^,^37^. These data suggest that the subtypes of NAcc response may constitute a trait-like property that is distributed throughout the general population.

Although the variable NAcc response patterns are not specific to depression, they may nevertheless be depression-related. Studies have demonstrated that on average NAcc activation differs between healthy and MDD groups not only in response to rewarding stimuli^14^,^16^,^19^,^27^ but also in response to other types of positively-valenced emotional stimuli^51^,^52^. Taken together, these results suggest that NAcc activity could be related to the vulnerability to or development of depression symptoms.

We thus extracted symptoms that were associated with the NAcc subtypes for MDD subjects. The results showed that NAcc response subtypes were indeed related to different symptoms (Table 3). Post-hoc analysis (Fig. 5) revealed that only the right NAcc response subtypes were significantly associated with suicidal ideation and anhedonia symptoms. We should mention that multivariate exploration analysis such as LDA with RFE in our analysis could aggressively extract any symptoms even if their association with the NAcc subtype was weak, so post-hoc analysis is important to confirm the validity and significance of the finding.

Right-side bias of depression-related neuropathology has often been reported^24^,^44^. Greenberg *et al.*^24^ showed abnormal reward expectation (RE) and prediction error (PE) signals only in the right ventral striatum. They also reported that group averages of HC and MDD either for the RE or PE signal were not significantly different, but distribution of individuals in RE × PE relationship (correlation between RE and PE) were different between the groups. This demonstrated the importance of investigating not only group averages but also individual variability to elucidate a neuropathology of depression.

Subjects in both hyper- and suppressed-activity subtypes for the right NAcc had greater severity in suicidal ideation and anhedonia symptoms. This result indicated that different types of neural activities could be associated with the same symptom. We also note that there was no significant difference in general symptom severity (sum score of multiple symptom scales) between subtypes (supplementary Table S7g-j). The association of both suicidal ideation and anhedonia appears consistent with reports indicating that anhedonia is a significant predictive risk factor for future suicide attempts^53^. Notably, our sensitivity to detect such a relationship may have been reduced by the exclusion of volunteers who presented a high risk for imminent suicidal behavior during screening.

Regarding anhedonia symptoms, while the association between the suppressed-activity and greater anhedonic symptom is convincing, the association between the hyperactive subtype and anhedonia symptom reported here appears counter-intuitive. This finding may conceivably be related to the reversed response patterns between anticipation and outcome periods. Subjects with hyperactive response to the anticipation of reward showed lower response at reward outcome (Fig. 3). This reversed response pattern appears consistent with the reinforcement learning model based on animal studies of dopamine neurons in the VTA and NAcc^54^. Changes in the electrophysiological responses of VTA and NAcc neurons can represent errors between the predicted value of reward or punishment and actual outcome value, so that unreasonably high expectations could result in reduced hedonic response during receipt of rewards. However, in the current results, the NAcc response of the hyperactive subtype at reward outcome was not significantly different from the other subtypes, while it was lower than the other subtypes. This hypothesis, therefore, was not confirmed in the current results. The reason that only one SHAPS item showed significant association with NAcc subtype is also not clear in the current results. As anhedonia is not a monolithic symptom and SHAPS cannot evaluate whole spectrum of anhedonia^8^, we might need further development of symptom measures to sort out various types of anhedonia.

Several limitations of the current study merit comment. While we focused on the NAcc response variability in gain and loss anticipations, this variability alone cannot explain the variance across all depressive phenotypes. Combined responses in both anticipation and outcome phase might elucidate another variability that may characterize MDD^24^. Thus, the same methodology should be applied to other neuroimaging paradigms and brain regions to account for subjects’ individual variations. To further elucidate the association between neuropathology and psychiatry symptoms, we also need to investigate transdiagnostic groups. Our proposed methodology, which elucidates subject variability independently of diagnosis, is definitely compatible with transdiagnostic study design and data analysis. The effect of sample size should also be considered in searching for subtypes. For example, since the number of subjects from a particular group in some subtypes was relatively small, our sensitivity was reduced for detecting a true difference in the relative proportions of subtypes between groups. We should also note that while we extracted clusters that showed statistically significant difference between groups, these clusters were not discrete groups. The cluster division was along with the first principal component (Fig. 2c) and subjects were continuously distributed in this axis (Fig. 2b). The NAcc response subtypes, therefore, were not distinctive groups but continuously distributed throughout the population.

In summary, we elucidated variations of NAcc responses to monetary gain and loss using an unsupervised brain subtyping and symptoms association analysis independent of DSM diagnosis. For MDD subjects, subtypes of NAcc responses were associated with anhedonia and suicidal ideation. Our novel study approach that identified variability in brain activations and then symptoms associated with the variations provides a useful novel strategy for elucidating pathophysiology heterogeneity associated with neuropsychiatric disorders.

## Methods

### Participants

Forty-four individuals with MDD (32 female) and 45 healthy control (HC) individuals (33 female) who had no personal history of a psychiatric disorder and no family history (in first degree relatives) of a mood disorder participated in the study. The study was approved by the Western Institutional Review Board, Puyallup, WA. Human research in this study was conducted according to the principles expressed in Declaration of Helsinki. All subjects gave written informed consent to participate in the study and received financial compensation.

The psychiatric diagnosis was established according to Diagnostic and Statistical Manual of Mental Disorders, Fourth Edition, Text Revision (DSM-IV-TR)^55^ criteria using both the Structural Clinical Interview for DSM-IV disorders administered via a trained clinical interviewer and an unstructured interview with a psychiatrist. Exclusion criteria included serious suicidal ideation, psychosis, major medical or neurological disorders, current pregnancy, general MRI exclusions, exposure to psychotropic medications or to any medication likely to influence cerebral function or blood flow within three weeks (8 weeks for fluoxetine), and meeting DSM-IV criteria for drug/alcohol abuse within the previous one year or for an alcohol/drug dependence (excepting nicotine) within the lifetime. Additional exclusion criteria applied to the HCs were current or past history of axis I psychiatric conditions either personally or in first degree relatives, as assessed using the Family Interview for Genetics Studies (FIGS)^56^.

### Experimental design of monetary incentive delay task

Supplementary Fig. S1 shows a procedure of the monetary incentive delay task. Five conditions composed of the following win/loss contingencies: −$1.0, −$0.25, $0, + $0.25, + $1.0, were applied in the task. One session consisted of 75 trials (15 trials for each of five conditions) and two sessions were performed. Before the experimental session, a practice session with 25 trials (5 for each condition) was performed in the scanner. The response time during the practice session was used to adjust the target duration. The target duration was set for a hit rate around 66%. The target duration was also adjusted during the experimental session by reducing one frame (1/75 s) after two consecutive hits and increasing one frame after one failure to keep the hit rate around 66% throughout the session.

### MRI measurement

Imaging was conducted on a whole-body 3 tesla MRI scanner (Discovery MR750, GE Healthcare, Milwaukee, WI) equipped with a 32-channel receive-only head array coil (Nova medical, Wilmington, MA). A single-shot gradient-recalled echo-planner imaging (EPI) sequence with sensitivity encoding (SENSE) was used for fMRI in the MID task session. The EPI imaging parameters were TR = 2000 ms, TE = 30 ms, FA = 90°, FOV = 240 mm, 37 axial slices with 3.0 mm thickness and 0.2 mm gap, matrix = 96 × 96, SENSE acceleration factor R = 2, number of volumes = 272, and scan time = 9 m 4 s. The EPI images were reconstructed into a 128 × 128 matrix resulting 1.875 × 1.875 × 3.2 mm^3^ voxel volume. A T1-weighted image with magnetization-prepared rapid gradient-echo (MPRAGE) sequence (FOV = 240 × 192 mm, matrix = 256 × 256, 120 axial slices, slice thickness = 0.9 mm, 0.9375 × 0.9375 × 0.9 mm^3^ voxel volume, TR = 5 ms, TE = 2.0 ms, R = 2, flip angle = 8°, delay time = 1400 ms, inversion time = 725 ms, sampling bandwidth = 31.2 kHz, scan time = 5 min 40 s) was acquired to provide anatomical reference for fMRI data.

### MRI data processing

Analysis of Functional NeuroImages (AFNI) software^57^ (http://afni.nimh.nih.gov/afni/) was used for fMRI data analysis. The first five volumes before starting the first trial in each session were excluded from analysis. Functional images underwent despiking, slice-timing correction, and motion correction by aligning to the first volume. The anatomical image was registered to the first functional volume and then was spatially normalized to the MNI template brain using Advanced Normalization Tools (ANTs, http://picsl.upenn.edu/software/ants/)^58^. The target template brain was resampled to 1.875 mm^3^ isotropic voxel. The nonlinear warping parameters estimated for the registered and resampled anatomical image were used to normalize the functional images. As a result, the voxel volume of the normalized functional image was 1.875 mm^3^ isotropic. Spatial smoothing was applied by convolving a 4.0 mm full width at half maximum (FWHM) Gaussian kernel. The signal time course was scaled to percent change relative to the mean signal across time in each voxel.

General linear model (GLM) analysis was conducted to evaluate hemodynamic brain activation. The design matrix included modeled responses for the delay period activation with variable duration boxcar function for each of the five conditions, the target onset and button press event with a delta function, and the feedback duration for each of the five conditions with a boxcar functions. Hit and miss trials were modeled separately for the feedback duration. These response models were convolved with a hemodynamic response function. Six motion parameters, their temporal derivatives, 4th-order polynomial regressors, and mean time-course of cerebrospinal fluid region were also included in the design matrix as noise regressors.

Beta coefficients for the delay period regressors at gain and loss conditions contrasted with the non-monetary condition were extracted as estimates of brain activation during anticipating gains and losses. Estimate of brain activation to gain outcomes was extracted by the contrast of hit trials in the reward condition to hit trials in the non-monetary condition. Estimate of brain activation to loss outcomes was extracted by the contrast of miss trials in the loss condition to miss trials in the non-monetary condition.

### NAcc region of interest analyses

Average responses in the left and right NAcc regions for each of four monetary conditions contrasted to the non-monetary condition were calculated in each subject. The NAcc mask was extracted from a FreeSurfer 5.3 (http://freesurfer.net/) segmentation map for the MNI template brain.

Principal component analysis was applied to the NAcc responses to elucidate response variation across subjects. Standard error of explained variance percentage was evaluated by the Jackknife resampling and principal scores were evaluated by leave-one-out cross-validation. Hierarchical clustering analysis was used to identify subtypes of NAcc activations to gain and loss anticipations. The analysis was applied to all subjects independent of diagnostic groups. Average responses in the left and right NAcc regions for each of four monetary conditions (contrasts with the non-monetary condition) were calculated in each subject. Similarity of NAcc activations between subjects was calculated by Euclidian distance between vectors of response pattern. Cluster tree was built by Ward’s method. An automatic cluster cut algorithm^59^ was applied to extract clusters (see Supplementary Fig. S2 for details). This algorithm extracts clusters in multiple levels and LMM analysis was used to find an optimal level. The level with significant main effect of cluster and with the minimum Bayesian information criterion value in the LMM analysis was taken as the optimal level to extract clusters.

### Extracting symptom items associated with the NAcc response subtypes

Twenty-one items in the Hamilton rating scale for depression (HAM-D)^45^, fourteen items in the Hamilton anxiety rating scale (HAM-A)^46^, ten items in the Montgomery-Asberg Depression Rating Scale (MADRS)^47^, and fourteen items of the Snaith-Hamilton Pleasure Scale (SHAPS)^48^ were used for symptom scores. The scores of each item were used as individual variables, which yielded fifty-nine symptom variables in the LDA analysis. For the SHAPS we used the range of 1–4 for scoring each item^60^ rather than the original 0–1 scoring range. This analysis was performed only for the MDD subjects since there were no or very small variances in these scores for the HC group.

To extract critical symptom items that characterize the NAcc response subtypes and to compensate for over-fitting problem in LDA, we used shrinkage discriminant analysis (SDA)^49^ combined with recursive feature elimination (RFE)^50^. Shrinkage regularization, which shrinks the off-diagonal values of the estimated covariance matrix toward zero, could reduce over-fitting risk when the number of variables is larger than the sample size, which was the case in our analysis using 59 symptoms for 44 MDD subjects. We used the ‘sda’ package in the R statistical computing language and environment. SDA learns a discriminant function LD(k) for each class k whose output is proportional to the log posterior probability of a class. RFE is a feature selection method in which unimportant variables for classification are eliminated step by step. A correlation-adjusted *t*-value (cat) score, which measures the individual contribution of each variable to separate groups after removing the effect of all other variables, was used to eliminate the unimportant variables^49^. One variable with the lowest cat score was removed at each RFE step. The cat scores were re-evaluated after eliminating a variable. Generalization performance at each elimination step was evaluated by a leave-one-out cross-validation. The symptom set that achieved the best generalization score was extracted. Note that the evaluated classification performance could be overestimated since we picked the best performance during RFE. The aim of this analysis, however, was not to estimate prediction accuracy from symptoms to NAcc subtypes, but to extract symptom subspace related to the NAcc subtypes.

### Statistical analysis

The LMM analysis was used for statistical tests of behavioral responses, symptom scores, and NAcc region of interest responses. The LMM analysis was performed with the R statistical computing language and environment nlme package^41^. Tukey’s test was used as a post-hoc test of LMM analysis. Reported *P*-values for post-hoc tests were corrected values.

## Additional Information

**How to cite this article**: Misaki, M. *et al.* Individual Variations in Nucleus Accumbens Responses Associated with Major Depressive Disorder Symptoms. *Sci. Rep.* **6**, 21227; doi: 10.1038/srep21227 (2016).

## Supplementary Material

Supplementary Information

## Figures and Tables

**Table 1:** Gender composition, age, and symptom rating scale scores for the HC and MDD subjects.

	HC	MDD	
N (male/female)	45 (33/12)	44 (32/12)	gender difference *P* = 1.000
Age (range, mean ± SD)	21–55, 32.0 ± 9.3	20–55, 35.3 ± 11.1	*t*(83.9) = 1.524*, P* = 0.131
HAM-D	2.6 ± 2.3	17.3 ± 5.6	*F*(1,85) = 264.200, *P* < 0.001
HAM-A	3.0 ± 3.1	17.8 ± 5.9	*F*(1,85) = 229.921, *P* < 0.001
MADRS	2.5 ± 2.8	23.0 ± 6.8	*F*(1,85) = 351.676, *P* < 0.001
SHAPS	18.3 ± 4.3	28.9 ± 6.2	*F*(1,82) = 85.667, *P* < 0.001

The result of Fisher’s exact test for gender composition, Welch’s two-sample *t*-test (two-tailed) for age, and main effect of diagnosis in linear-mixed effect model analysis with fixed effect of diagnosis, age, and gender are shown in the table.

HC: healthy control. MDD: major depressive disorder. HAM-D: Hamilton rating scale for depression-21 item. HAM-A: Hamilton anxiety rating scale. MADRS: Montgomery-Asberg Depression Rating Scale. SHAPS: Snaith-Hamilton Pleasure Scale (higher total SHAPS score indicated higher levels of present state anhedonia).

**Table 2:** Results of the linear mixed-effect model analysis for nucleus accumbens responses during anticipating gain or loss.

Factor	Left NAcc			Right NAcc		
	DFs	*F*	*P*	DFs	*F*	*P*
Subtype	3, 79	95.465	<0.001	2, 81	91.897	<0.001
Condition	3, 243	41.845	<0.001	3, 249	35.183	<0.001
Diagnosis	1, 79	0.101	0.752	1, 81	0.126	0.723
Age	1, 79	0.076	0.784	1, 81	0.022	0.883
Gender	1, 79	0.000	0.986	1, 81	0.080	0.778
Subtype × Condition	9, 243	10.506	<0.001	6, 249	9.653	<0.001
Subtype × Diagnosis	3, 79	0.597	0.619	2, 81	0.129	0.879
Condition × Diagnosis	3, 243	1.754	0.157	3, 249	1.886	0.132
Subtype × Condition × Diagnosis	9, 243	0.666	0.740	6, 249	3.517	0.002

The model included fixed effects of response subtype, monetary condition, diagnosis, age, and gender and random effect of subject.

NAcc = nucleus accumbens, DFs = degrees of freedom

**Table 3:** List of symptom items selected for discriminating NAcc response subtypes and their correlations with the output of the linear discriminant function (LD) for (a) left NAcc and (b) right NAcc.

**a.**				
**Symptom**	**LD(A)**	**LD(B)**	**LD(C)**	**LD(D)**
**Insomnia (HAM-A)**	0.024	−0.263	0.566**	−0.338
**Reduced sleep (MADRS)**	0.019	−0.164	0.471*	−0.334
**Depersonalization and Derealization (HAM-D)**	−0.375	−0.262	−0.119	0.660**
Severity of diurnal variation (HAM-D)	0.450	−0.147	0.221	−0.422
Somatic (Muscular) (HAM-A)	−0.442	−0.214	0.208	0.332
I would get pleasure from helping others (SHAPS)	0.432	0.222	−0.174	−0.366
Anxiety Somatic (HAM-D)	−0.372	−0.266	0.095	0.443
enjoy seeing other people’s smiling faces (SHAPS)	0.238	0.344	−0.282	−0.227
Somatic (Sensory) (HAM-A)	−0.181	−0.389	0.376	0.132
Autonomic Symptoms (HAM-A)	−0.340	−0.201	0.057	0.395
Concentration difficulties (MADRS)	0.272	0.233	−0.258	−0.170
I would find pleasure in my hobbies or pastimes (SHAPS)	0.251	0.069	−0.396	0.146
Insomnia Late (Early Awakening) (HAM-D)	0.046	−0.191	0.366	−0.222
I would enjoy a warm bath or refreshing shower (SHAPS)	0.388	−0.178	0.046	−0.168
Retardation (HAM-D)	−0.083	0.344	−0.170	−0.098
Insomnia Early (Sleep Onset Insomnia) (HAM-D)	0.143	0.033	0.176	−0.319
I would enjoy being with my family or close friends (SHAPS)	0.191	0.043	−0.257	0.075
Loss of Weight (HAM-D)	−0.143	0.186	0.080	−0.155
be able to enjoy my favorite meal (SHAPS)	0.261	0.048	−0.240	0.000
Reported Sadness (MADRS)	−0.119	−0.169	0.077	0.176
Work and Activities (HAM-D)	0.289	−0.033	−0.031	−0.156
Apparent Sadness (MADRS)	0.018	−0.248	0.072	0.155
Obsessional and Compulsive Symptoms (HAM-D)	0.032	0.219	−0.042	−0.194
Fears (HAM-A)	−0.006	−0.185	−0.027	0.212
Inner tension (MADRS)	−0.237	0.090	0.076	0.014
**b.**				
**Symptom**	**LD(A)**	**LD(B)**	**LD(C)**	
Suicidal ideation (HAM-D)	0.653**	−0.811**	0.539**	
I would enjoy looking smart when I have made an effort with my appearance (SHAPS)	0.606**	−0.748**	0.494**	
Behavior at Interview (HAM-A)	0.311	−0.522**	0.477*	
Genital Symptoms (HAM-D)	0.177	−0.488**	0.582**	
Somatic (Sensory) (HAM-A)	−0.698**	0.459*	0.084	
Somatic (Muscular) (HAM-A)	−0.418*	−0.021	0.530**	
Insomnia Late (Early Awakening) (HAM-D)	−0.302	−0.059	0.453*	
I would get pleasure from helping others (SHAPS)	0.373	−0.144	−0.208	
Loss of Weight (HAM-D)	−0.190	0.223	−0.137	

Items having a significant correlation after Bonferroni correction with a NAcc subtype discriminant function indicated by bold font, were placed at the top of the list. The other items are ordered by their average absolute correlation value in descending order. *P < 0.05, **P < 0.01 (corrected).
