# Supplementary material for: Individual Variations in Nucleus Accumbens Responses Associated with Major Depressive Disorder Symptoms
Source: Sci Rep. 2016 Feb 16;6:21227. doi: 10.1038/srep21227 (PMC4754797; doi:10.1038/srep21227)
Supplement: Supplementary Information [file srep21227-s1.pdf]

Supplementary materials for “Individual Variations in Nucleus Accumbens Responses Associated with Major Depressive Disorder Symptoms”

Masaya Misaki, Hideo Suzuki, Jonathan Savitz, Wayne C. Drevets, and Jerzy Bodurka

Figure S1

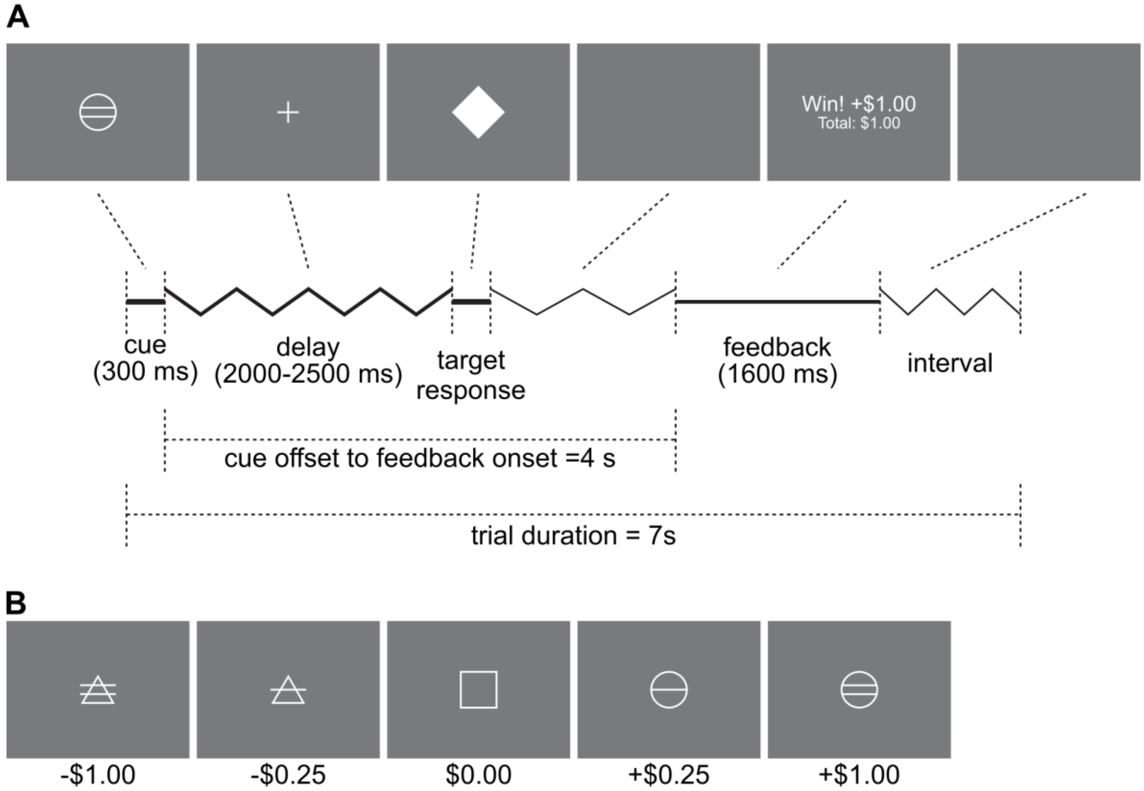

Diagram of monetary incentive delay (MID) task procedure. (A) Sequence and timing of one trial. (B) Cue patterns indicating the trial conditions.

Figure S2

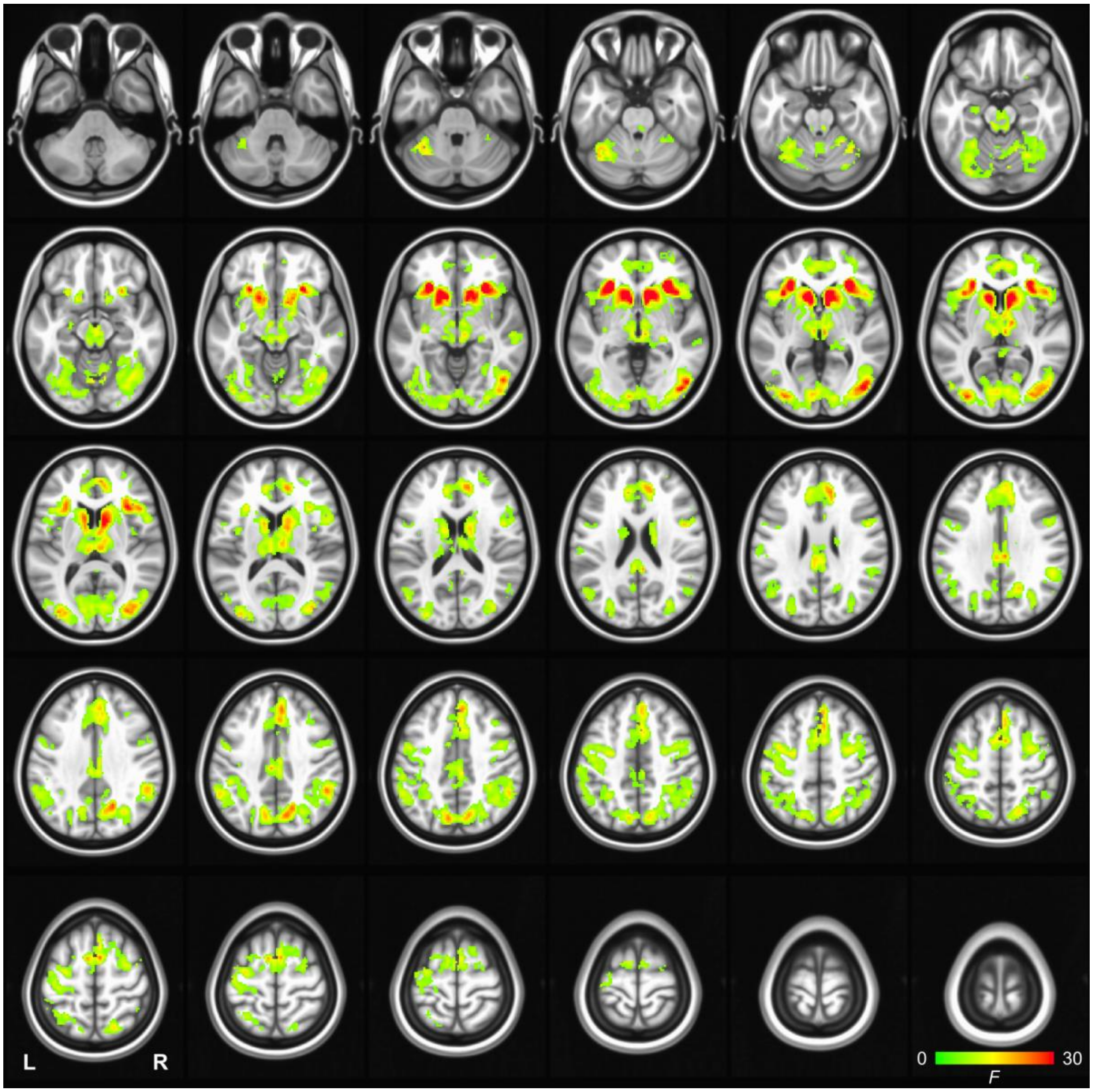

Whole brain *F*-value map of the main effect of monetary condition in the LME analysis for the response in the anticipation period with condition, diagnosis (HC, MDD), condition x diagnosis interaction, age, and gender as fixed effects and subject as a random effect. The axial slices from z = -38 mm to +78 mm with 4 mm interval were shown with *F*-value overlay. The map was thresholded by uncorrected $P < 0.001$ and cluster size > 43, which corresponds to $P < 0.05$ with the Monte Carlo simulation of 3dClustSim in AFNI.

Figure S3

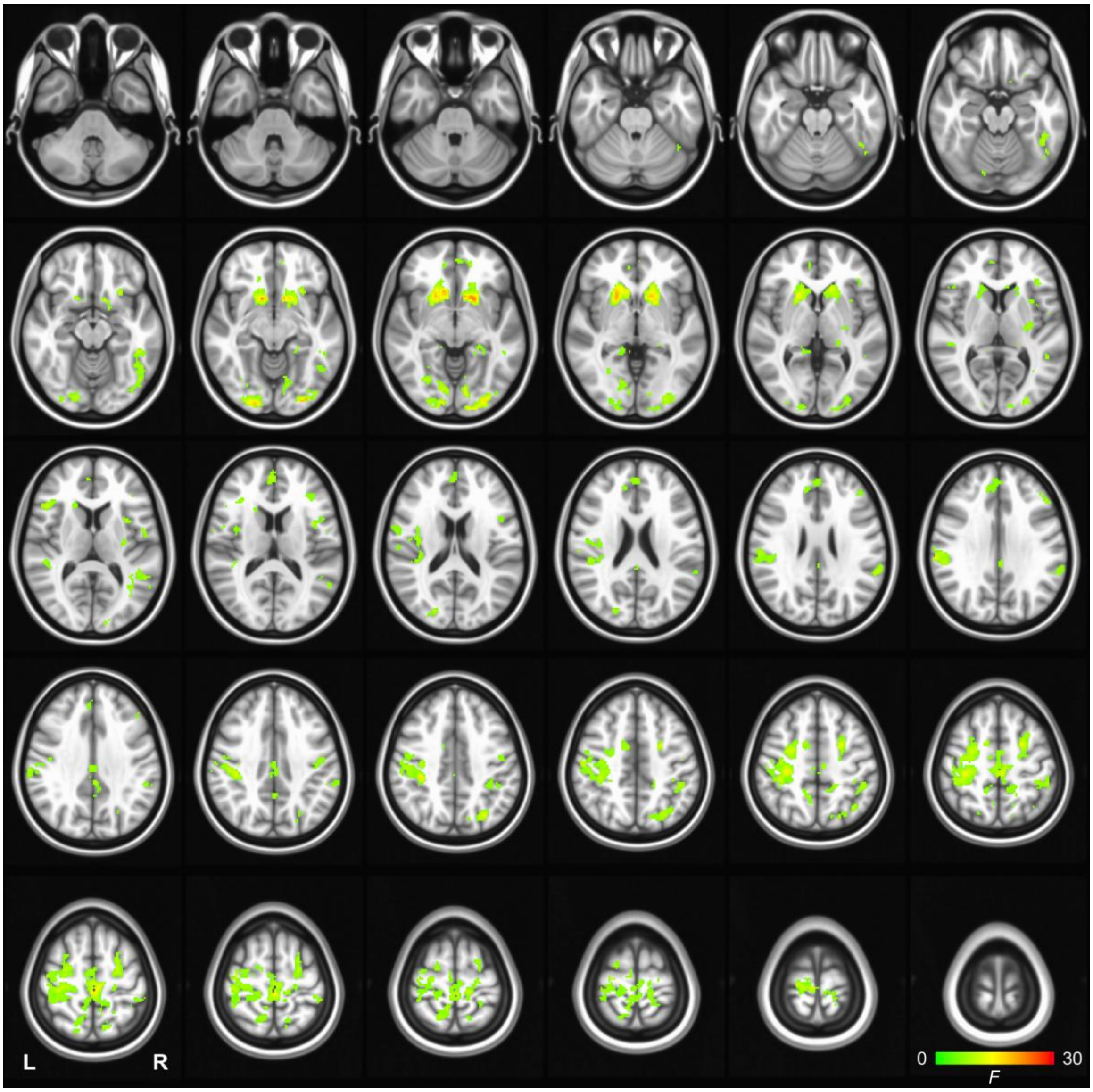

Whole brain *F*-value map of the main effect of monetary condition in the LME analysis for the response in the outcome period with condition, diagnosis (HC, MDD), condition x diagnosis interaction, age, and gender as fixed effects and subject as a random effect. The axial slices from z = -38 mm to +78 mm with 4 mm interval were shown with *F*-value overlay. The map was thresholded by uncorrected $P < 0.001$ and cluster size > 43, which corresponds to $P < 0.05$ with the Monte Carlo simulation of 3dClustSim in AFNI.

Table S1
LME analysis for the NAcc responses in the anticipation (A) and outcome period (B).

A. Anticipation period

		**Left NAcc**		**Right NAcc**	
**Factor**	**DFs**	***F***	***P***	***F***	***P***
Condition	3, 261	32.260	< 0.001	28.391	< 0.001
Diagnosis	1, 85	3.077	0.083	0.982	0.325
Age	1, 85	2.252	0.137	5.041	0.027
Gender	1, 85	1.619	0.207	2.058	0.155
Condition x Diagnosis	3, 261	2.637	0.050	2.755	0.043

B. Outcome period

		**Left NAcc**		**Right NAcc**	
**Factor**	**DFs**	***F***	***P***	***F***	***P***
Condition	3, 261	12.777	< 0.001	10.764	< 0.001
Diagnosis	1, 85	2.321	0.131	0.699	0.405
Age	1, 85	4.202	0.044	4.551	0.036
Gender	1, 85	0.180	0.672	0.580	0.449
Condition x Diagnosis	3, 261	0.796	0.497	0.831	0.478

DFs = degrees of freedom

Figure S2

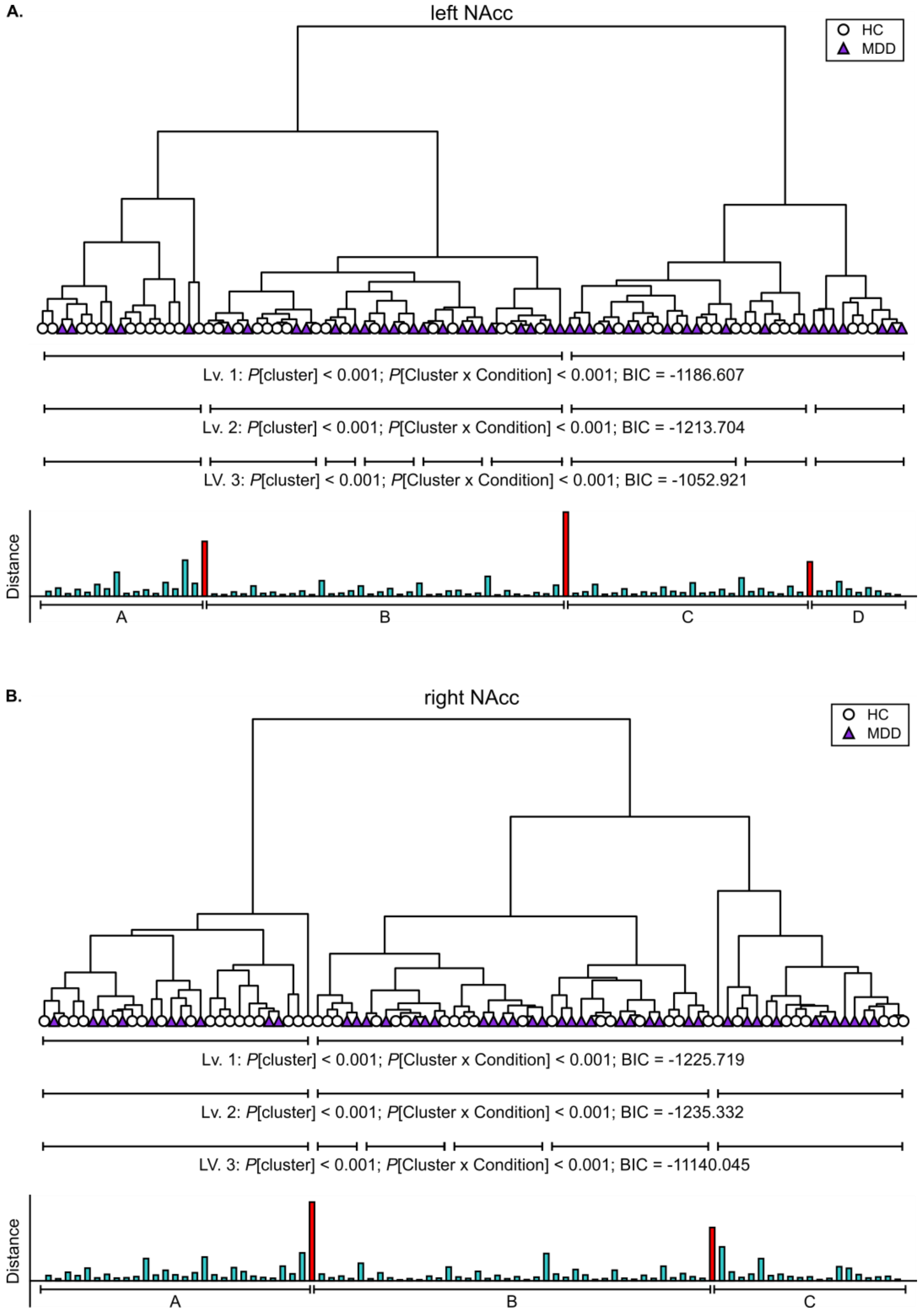

Clustering analysis for NAcc responses in the anticipation period. Dendrogram of clusters tree (top), extracted flat clusters by automatic cluster cut algorithm (Sander et al., 2003) (middle), and reachability distance plot (bottom) are shown for the left (A) and right (B)

NAcc response patterns. Reachability distance indicates path length on the tree to reach the right neighboring leaf node (Sander et al., 2003). Before calculating the length, each branch of the tree had been rotated for a right leaf node to be the nearest sample within the right tree. In the automatic cluster cut algorithm, cluster tree was cut at the path length that was 1.3 times longer than the average length within all branches under the point. This algorithm extracted multiple levels of flat clusters (Sander et al., 2003). Levels 1 to 3 are shown in the figure. To find the optimal level, the linear-mixed effect (LME) analysis was performed for each level with the NAcc response value as a dependent variable, condition, age, and gender as fixed effects and subject as a random effect. The level with a significant effect of cluster and with the minimum Bayesian information criterion (BIC) value was considered as the optimal level. BIC provides a model selection criterion, which evaluates the goodness of fit of the model with penalizing the complexity of the model. The lower BIC value indicates the better model. The main effect of cluster and its interaction with the task condition were significant at all levels. BIC value was minimum at the level 2 for both left and right NAcc. We took this level of clusters as the subtypes of NAcc responses.

Table S2

Number of subjects in the left and right subtypes

		Right NAcc		
		A	B	C
Left NAcc	A	15	2	0
	B	13	21	3
	C	0	15	10
	D	0	3	7

Association between the left and right NAcc subtypes was significant by chi-square test. ($\chi^2(6) = 56.149$, $P<0.001$).

Table S3

Post-hoc test (Tukey's test) of the LME analysis for the left NAcc responses during anticipation of reward and loss. Tables A to D show the results of multiple comparisons test between conditions in each subtype. Upper triangle cells indicate *t*-values and lower triangle cells indicate *P*-values. Degree of freedom for the comparisons between gain and loss conditions is 243 and for the comparisons with the $0 conditions is 79.

A. Multiple comparisons between conditions in the left subtype A

Conditions	-$1.0	-$0.25	+$0.25	+$1.0	$0
**-$1.0**		3.886	3.169	-5.696	11.763
**-$0.25**	0.001		-0.718	-9.582	7.335
**+$0.25**	0.009	0.890		-8.864	8.153
**+$1.0**	<0.001	<0.001	<0.001		18.253
**$0**	<0.001	<0.001	<0.001	<0.001	

B. Multiple comparisons between conditions in the left subtype B

Conditions	-$1.0	-$0.25	+$0.25	+$1.0	$0
**-$1.0**		-0.505	-1.496	-5.481	5.985
**-$0.25**	0.958		-0.991	-4.976	6.555
**+$0.25**	0.442	0. 755		-3.986	7.673
**+$1.0**	<0.001	<0.001	0.001		12.173
**$0**	<0.001	<0.001	<0.001	<0.001	

C. Multiple comparisons between conditions in the left subtype C

Conditions	-$1.0	-$0.25	+$0.25	+$1.0	$0
**-$1.0**		1.979	-0.634	-0.240	1.113
**-$0.25**	0.199		-2.613	-2.219	-1.007
**+$0.25**	0.921	0.047		0.394	1.793
**+$1.0**	0.995	0.121	0.979		1.371
**$0**	0.269	0.317	0.077	0.174	

D. Multiple comparisons between conditions in the left subtype D

Conditions	-$1.0	-$0.25	+$0.25	+$1.0	$0
**-$1.0**		-1.207	-2.752	-4.933	-5.652
**-$0.25**	0.623		-1.544	-3.726	-4.284
**+$0.25**	0.032	0.413		-2.182	-2.533
**+$1.0**	<0.001	0.001	0.131		-0.061
**$0**	<0.001	<0.001	0.013	0.952	

Table S4

Post-hoc test (Tukey's test) of the LME analysis for the left NAcc responses during outcome of reward and loss. Conventions are the same as in Tab. S3. Degree of freedom for the comparisons between gain and loss conditions is 243 and for the comparisons with the $0 conditions is 79.

A. Multiple comparisons between conditions in the left subtype A

Conditions	-$1.0	-$0.25	+$0.25	+$1.0	$0
**-$1.0**		-2.646	-5.006	-3.662	-5.069
**-$0.25**	0.043		-2.360	-1.016	-1.847
**+$0.25**	<0.001	0.088		1.344	1.027
**+$1.0**	0.002	0.740	0.536		-0.610
**$0**	<0.001	0.069	0.308	0.544	

B. Multiple comparisons between conditions in the left subtype B

Conditions	-$1.0	-$0.25	+$0.25	+$1.0	$0
**-$1.0**		-0.900	-2.621	-1.658	-1.808
**-$0.25**	0.805		-1.721	-0.758	-0.720
**+$0.25**	0.046	0.315		0.963	1.359
**+$1.0**	0.348	0.873	0.770		0.195
**$0**	0.074	0.473	0.178	0.846	

C. Multiple comparisons between conditions in the left subtype C

Conditions	-$1.0	-$0.25	+$0.25	+$1.0	$0
**-$1.0**		-2.519	-2.730	-3.265	-3.261
**-$0.25**	0.059		-0.211	-0.745	-0.353
**+$0.25**	0.034	0.997		-0.534	-0.110
**+$1.0**	0.007	0.879	0.951		0.507
**$0**	0.002	0.725	0.913	0.614	

D. Multiple comparisons between conditions in the left subtype D

Conditions	-$1.0	-$0.25	+$0.25	+$1.0	$0
**-$1.0**		-1.007	-0.395	-2.131	0.146
**-$0.25**	0.745		0.612	-1.124	1.367
**+$0.25**	0.979	0.928		-1.736	0.625
**+$1.0**	0.146	0.675	0.307		2.730
**$0**	0.884	0.176	0.534	0.008	

Table S5

Post-hoc test (Tukey's test) of the LME analysis for the left NAcc responses during anticipation of reward and loss. Conventions are the same as in Tab. S3. Degree of freedom for the comparisons between gain and loss conditions is 249 and for the comparisons with the $0 conditions is 81.

A. Multiple comparisons between conditions in the right subtype A

Conditions	-$1.0	-$0.25	+$0.25	+$1.0	$0
**-$1.0**		2.843	1.572	-5.606	9.811
**-$0.25**	0.025		-1.271	-8.448	6.574
**+$0.25**	0.397	0.582		-7.177	8.021
**+$1.0**	<0.001	<0.001	<0.001		16.196
**$0**	<0.001	<0.001	<0.001	<0.001	

B. Multiple comparisons between conditions in the right subtype B

Conditions	-$1.0	-$0.25	+$0.25	+$1.0	$0
**-$1.0**		-0.898	-3.892	-6.114	1.358
**-$0.25**	0.806		-2.994	-5.216	2.357
**+$0.25**	0.001	0.016		-2.222	5.690
**+$1.0**	<0.001	<0.001	0.120		8.164
**$0**	0.178	0.021	<0.001	<0.001	

C. Multiple comparisons between conditions in the right subtype C

Conditions	-$1.0	-$0.25	+$0.25	+$1.0	$0
**-$1.0**		-0.077	1.078	-0.796	-3.058
**-$0.25**	1.000		1.154	-0.719	-2.974
**+$0.25**	0.704	0.656		-1.873	-4.247
**+$1.0**	0.856	0.889	0.242		-2.180
**$0**	0.003	0.004	<0.001	0.032	

Table S6

Post-hoc test (Tukey’s test) of the LME analysis for the left NAcc responses during outcome of reward and loss. Conventions are the same as in Tab. S3. Degree of freedom for the comparisons between gain and loss conditions is 249 and for the comparisons with the $0 conditions is 81.

A. Multiple comparisons between conditions in the right subtype A

Conditions	-$1.0	-$0.25	+$0.25	+$1.0	$0
**-$1.0**		-1.359	-3.764	-2.710	-4.667
**-$0.25**	0.527		-2.406	-1.352	-2.998
**+$0.25**	0.001	0.079		1.054	-0.043
**+$1.0**	0.036	0.531	0.718		-1.338
**$0**	<0.001	0.004	0.966	0.185	

B. Multiple comparisons between conditions in the right subtype B

Conditions	-$1.0	-$0.25	+$0.25	+$1.0	$0
**-$1.0**		-1.793	-3.570	-3.451	-2.518
**-$0.25**	0.279		-1.778	-1.658	-0.358
**+$0.25**	0.002	0.287		0.119	1.784
**+$1.0**	0.004	0.348	0.999		1.641
**$0**	0.014	0.722	0.078	0.105	

C. Multiple comparisons between conditions in the right subtype C

Conditions	-$1.0	-$0.25	+$0.25	+$1.0	$0
**-$1.0**		-0.113	-0.152	-0.996	-0.137
**-$0.25**	1.000		-0.039	-0.884	-0.003
**+$0.25**	0.999	1.000		-0.845	0.044
**+$1.0**	0.752	0.813	0.833		1.054
**$0**	0.891	0.998	0.965	0.295	

Table S7

Demographics, behavioral responses in the MID task, and symptom ratings for NAcc response subtypes. (A) Gender composition, (B) age, (C) socioeconomic status, (D-F) behavioral responses in the MID task; (D) mean reaction time, (E) hit rate, (F) total earned money, (G) HAM-D, (H) HAM-A, (I) MADRS, (J) SHAPS, (K) number of depressed episodes, (L) years since the first episode. Gender composition was tested by chi-square test. Other variables were tested by a linear mixed-effect (LME) model analysis with listed fixed effects.

A. Gender composition

	Left NAcc				Right NAcc		
**Subtype**	**A**	**B**	**C**	**D**	**A**	**B**	**C**
**N (female/male)**	12/5	25/12	20/5	8/2	19/9	31/10	15/5
**Chi-square test**	$\chi^2(3)=1.475$, $P=0.688$				$\chi^2(2)=0.558$, $P=0.756$		

B. Age

	Left NAcc				Right NAcc		
**Subtype**	**A**	**B**	**C**	**D**	**A**	**B**	**C**
**Mean**	32.9	30.2	37.9	36.4	30.5	33.1	38.9
**SD**	9.7	8.6	11.1	12.2	9.1	10.6	9.8

LME analysis

	Left NAcc			Right NAcc		
**Factor**	**DFs**	***F***	***P***	**DFs**	***F***	***P***
Subtype	3,85	3.292	0.024	2,86	4.141	0.019

C. Socioeconomic status (Hollingshead Four Factor Index of Socioeconomic Status)

Fourteen subjects whose socioeconomic status was not available were excluded.

	Left NAcc				Right NAcc		
**Subtype**	**A**	**B**	**C**	**D**	**A**	**B**	**C**
**N**	15	29	22	9	23	33	19
**Mean**	163.1	133.5	102.4	95.9	151.4	101.0	137.7
**SD**	175.0	162.9	138.9	135.0	169.2	139.0	163.2

LME analysis

	Left NAcc			Right NAcc		
**Factor**	**DFs**	***F***	***P***	**DFs**	***F***	***P***
Subtype	3,69	0.597	0.619	2,70	0.820	0.445
Age	1,69	1.542	0.219	1,70	2.295	0.134
Gender	1,69	1.786	0.186	1,70	2.057	0.156

D. Reaction time in MID task

	Left NAcc				Right NAcc		
**Subtype**	**A**	**B**	**C**	**D**	**A**	**B**	**C**
**Mean (ms)**	301.3	326.1	335.1	325.8	300.8	331.2	341.3
**SD**	170.3	186.7	179.4	173.9	169.2	188.7	175.8

LME analysis

	Left NAcc			Right NAcc		
**Factor**	**DFs**	***F***	***P***	**DFs**	***F***	***P***
Subtype	3,82	2.262	0.087	2,83	6.612	0.002
Diagnosis	1,82	4.032	0.048	1,83	3.133	0.080
Condition	4,13106	23.924	<0.001	4,13106	23.919	<0.001
Age	1,82	2.495	0.118	1,83	1.176	0.281
Gender	1,82	8.424	0.005	1,83	6.328	0.014

Post-hoc test for the right NAcc subtype showed no significant difference between subtypes: A-B, *P*=0.069; A-C, *P*=0.083; B-C, *P*=0.922

E. Hit rate in MID task

	Left NAcc				Right NAcc		
**Subtype**	**A**	**B**	**C**	**D**	**A**	**B**	**C**
**Mean (%)**	63.9	65.2	65.0	64.7	64.4	64.7	65.8
**SD**	2.6	2.7	3.1	2.1	2.5	2.6	3.2

LME analysis

	Left NAcc			Right NAcc		
**Factor**	**DFs**	***F***	***P***	**DFs**	***F***	***P***
Subtype	3,82	0.455	0.715	2,83	0.915	0.405
Diagnosis	1,82	0.184	0.669	1,83	0.229	0.633
Condition	4,13257	13.737	<0.001	4,13275	13.738	<0.001
Age	1,82	0.868	0.354	1,83	0.213	0.646
Gender	1,82	0.215	0.644	1,83	0.522	0.472

F. Total earned money in MID task

	Left NAcc				Right NAcc		
**Subtype**	**A**	**B**	**C**	**D**	**A**	**B**	**C**
**Mean ($)**	13.37	12.70	12.38	11.98	13.22	12.38	12.44
**SD**	2.93	3.33	3.58	3.39	3.10	3.53	3.18

LME analysis

	Left NAcc			Right NAcc		
**Factor**	**DFs**	***F***	***P***	**DFs**	***F***	***P***
Subtype	3,82	0.490	0.690	2,83	0.637	0.531
Diagnosis	1,82	9.057	0.004	1,83	8.755	0.004
Age	1,82	0.012	0.912	1,83	0.061	0.806
Gender	1,82	0.274	0.602	1,83	0.334	0.565

G. HAM-D

	Left NAcc				Right NAcc		
**Subtype**	**A**	**B**	**C**	**D**	**A**	**B**	**C**
**Mean**	16.6	17.2	18.2	17.0	17.1	16.2	19.9
**SD**	3.9	5.5	4.7	8.8	4.3	4.9	7.4

LME analysis

	Left NAcc			Right NAcc		
**Factor**	**DFs**	***F***	***P***	**DFs**	***F***	***P***
Subtype	3,38	0.126	0.944	2,39	1.638	0.207
Age	1,38	1.980	0.168	1,39	0.564	0.457
Gender	1,38	0.848	0.363	1,39	0.644	0.427

H. HAM-A

	Left NAcc				Right NAcc		
**Subtype**	**A**	**B**	**C**	**D**	**A**	**B**	**C**
**Mean**	16.0	17.3	18.7	19.4	15.7	17.5	20.5
**SD**	5.5	6.0	5.3	7.4	6.1	5.4	6.3

LME analysis

	Left NAcc			Right NAcc		
**Factor**	**DFs**	***F***	***P***	**DFs**	***F***	***P***
Subtype	3,38	0.474	0.702	2,39	1.837	0.173
Age	1,38	2.015	0.164	1,39	0.902	0.348
Gender	1,38	1.229	0.275	1,39	0.872	0.356

I. MADRS

	Left NAcc				Right NAcc		
**Subtype**	**A**	**B**	**C**	**D**	**A**	**B**	**C**
**Mean**	22.0	23.1	23.7	22.6	22.8	22.6	24.2
**SD**	4.5	7.1	5.8	9.9	5.7	6.3	9.1

LME analysis

	Left NAcc			Right NAcc		
**Factor**	**DFs**	***F***	***P***	**DFs**	***F***	***P***
Subtype	3,38	0.079	0.971	2,39	0.198	0.821
Age	1,38	0.128	0.722	1,39	0.009	0.927
Gender	1,38	2.170	0.150	1,39	1.872	0.179

J. SHAPS

Two MDD subjects who had missing values in SHAPS were excluded.

	Left NAcc				Right NAcc		
**Subtype**	**A**	**B**	**C**	**D**	**A**	**B**	**C**
**N**	5	20	11	6	10	21	11
**Mean**	32.6	29.4	26.3	29.2	31.2	27.7	29.2
**SD**	6.4	5.4	6.6	7.3	5.2	6.6	6.2

LME analysis

	Left NAcc			Right NAcc		
**Factor**	**DFs**	***F***	***P***	**DFs**	***F***	***P***
Subtype	3,36	1.291	0.292	2,37	1.078	0.352
Age	1,36	1.152	0.290	1,37	0.370	0.547
Gender	1,36	0.011	0.917	1,37	0.148	0.703

K. Number of depressed episodes

Eighteen MDD subjects whose number of depressed episodes was not available were excluded.

	Left NAcc				Right NAcc		
**Subtype**	**A**	**B**	**C**	**D**	**A**	**B**	**C**
**N**	4	11	7	4	8	12	6
**Mean**	8.0	7.7	5.5	10.0	7.1	6.2	10.8
**SD**	11.4	11.6	6.6	15.4	8.9	10.8	12.9

LME analysis

	Left NAcc			Right NAcc		
**Factor**	**DFs**	***F***	***P***	**DFs**	***F***	***P***
Subtype	3,20	0.135	0.938	2,21	0.354	0.706
Age	1,20	0.436	0.517	1,21	0.066	0.800
Gender	1,20	0.321	0.577	1,21	0.068	0.797

L. Years since the first episode

Twelve MDD subjects whose years since the first episode was not available were excluded.

	Left NAcc				Right NAcc		
**Subtype**	**A**	**B**	**C**	**D**	**A**	**B**	**C**
**N**	5	13	10	4	8	17	7
**Mean**	16.6	10.8	19.4	17.3	13.4	14.9	18.0
**SD**	5.6	3.6	15.8	14.7	5.7	12.2	12.6

LME analysis

	Left NAcc			Right NAcc		
**Factor**	**DFs**	***F***	***P***	**DFs**	***F***	***P***
Subtype	3,26	1.964	0.144	2,27	0.544	0.587
Age	1,26	14.430	0.001	1,27	18.794	<0.001
Gender	1,26	1.254	0.273	1,27	0.890	0.354

Figure S3

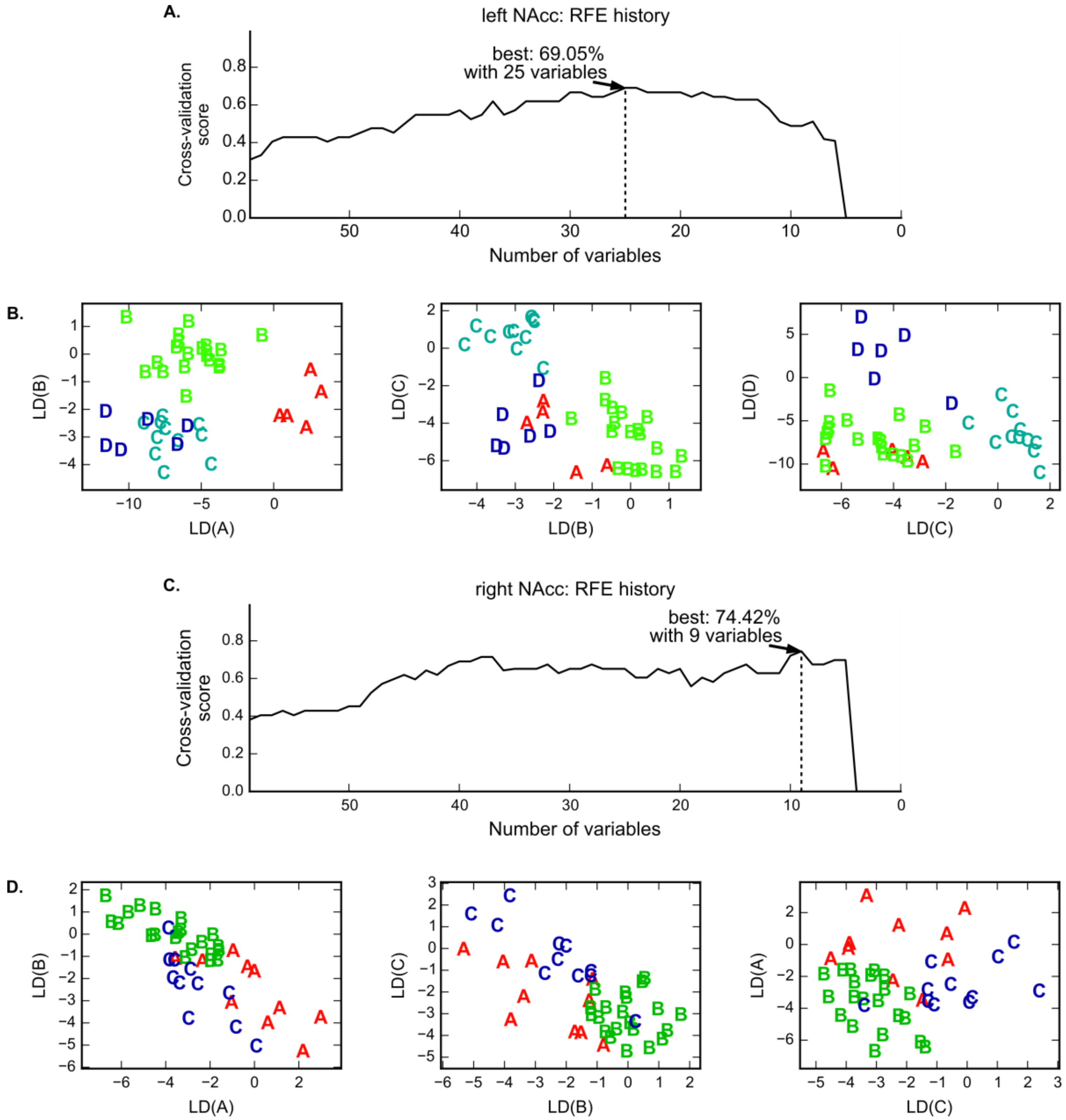

Linear discriminant analysis with recursive feature elimination (RFE) for the left (A, B) and right (C, D) NAcc subtypes with symptom scores. (A) History of leave-one-out cross-validation scores in the RFE for the left NAcc subtypes. The best classification score was achieved with a set of 25 variables. (B) Plots of discriminant function output for the left NAcc subtypes. Plotted character indicates subject's subtype.
(C) History of leave-one-out cross-validation scores in the RFE for the right NAcc subtypes. The best classification score was achieved with a set of nine variables. (D) Plots of discriminant function output for the right NAcc subtypes.

References

Sander, J., Qin, X., Lu, Z., Niu, N., Kovarsky, A., 2003. Automatic extraction of clusters from hierarchical clustering representations. Proceedings of the 7th Pacific-Asia conference on Advances in knowledge discovery and data mining. Springer-Verlag, Seoul, Korea, pp. 75-87.
